# Case report: VA-ECMO as a bridge to collapse in carcinoid heart disease

**DOI:** 10.1093/ehjcr/ytag272

**Published:** 2026-04-29

**Authors:** Ana Alicia Alañón Hernández, Javier Tobar Ruiz, Jose Francisco Gil Fernandez, Javier García de Casasola Rodriguez, Maria Plaza Martín

**Affiliations:** Department of Cardiology, Hospital Clínico Universitario de Valladolid, Avenida de Ramón y Cajal 3, 47003 Valladolid (Castilla y León), Spain; Department of Cardiology, Hospital Clínico Universitario de Valladolid, Avenida de Ramón y Cajal 3, 47003 Valladolid (Castilla y León), Spain; Centro de Investigación Biomédica en Red de Enfermedades Cardiovasculares (CIBERCV), 28029 Madrid, Spain; Instituto de Investigación Biomédica de Valladolid (IBioVAll), 47003 Valladolid, Spain; Department of Cardiology, Hospital Clínico Universitario de Valladolid, Avenida de Ramón y Cajal 3, 47003 Valladolid (Castilla y León), Spain; Department of Cardiology, Hospital Clínico Universitario de Valladolid, Avenida de Ramón y Cajal 3, 47003 Valladolid (Castilla y León), Spain; Department of Cardiology, Hospital Clínico Universitario de Valladolid, Avenida de Ramón y Cajal 3, 47003 Valladolid (Castilla y León), Spain; Centro de Investigación Biomédica en Red de Enfermedades Cardiovasculares (CIBERCV), 28029 Madrid, Spain

**Keywords:** Case report, Carcinoid heart disease, Veno-arterial extracorporeal membrane oxygenation (VA-ECMO), Mechanical circulatory support, Right ventricular failure, Neuroendocrine tumour

## Abstract

**Background:**

Carcinoid heart disease is a rare complication of neuroendocrine tumours caused by chronic exposure to vasoactive substances, leading to progressive valvular dysfunction and right heart failure. The use of veno-arterial extracorporeal membrane oxygenation (VA-ECMO) in this context remains controversial, as it may exacerbate carcinoid crisis by bypassing the pulmonary metabolism of these vasoactive mediators.

**Case summary:**

We describe a patient with clinical suspicion of a neuroendocrine tumour and severe carcinoid heart disease who developed refractory cardiogenic shock. VA-ECMO was initiated as rescue support but resulted in an abrupt haemodynamic collapse, the patient subsequently progressed to multiorgan failure and died. This case illustrates the paradoxical and potentially deleterious effects of extracorporeal support in the setting of carcinoid physiology.

**Discussion:**

In the setting of a carcinoid crisis with cardiogenic shock secondary to right ventricular failure, VA-ECMO may appear as a potential option for haemodynamic support. However, by reducing pulmonary blood flow—and consequently the degradation of vasoactive substances—its use may worsen the patient’s clinical condition. Therefore, a multidisciplinary approach involving cardiology, acute cardiac care, intensive care, oncology, gastroenterology, endocrinology, and pathology teams is essential, both in diagnosis and management.

Learning points

- This case highlights the importance of a physiology-based management strategy in carcinoid heart disease with cardiogenic shock.
- Therapeutic decisions must be guided not only by haemodynamic parameters but also by a thorough understanding of neuroendocrine pathophysiology.

## Introduction

Carcinoid heart disease (CHD) is a rare but severe presentation of neuroendocrine tumours. It is due to a prolonged exposure to vasoactive substances such as serotonin. This exposure leads to a progressive fibrotic involvement of the right-sided cardiac valves frequently resulting in severe valvular disease, right ventricular dysfunction, and advanced heart failure.

In the setting of cardiogenic shock, veno-arterial extracorporeal membrane oxygenation (VA-ECMO) is increasingly used as a rescue therapy. However, particularly in patients with carcinoid physiology, extracorporeal support may have paradoxical deleterious effects. As VA-ECMO bypass the pulmonary circulation—consequently reducing pulmonary blood flow—it limits the pulmonary metabolism of vasoactive mediators, potentially exacerbating carcinoid crisis and precipitating haemodynamic collapse. We report a case that highlights this interaction and reveals the importance of careful patient selection and multidisciplinary decision-making when considering VA-ECMO support in CHD.

## Summary figure

**Figure:**
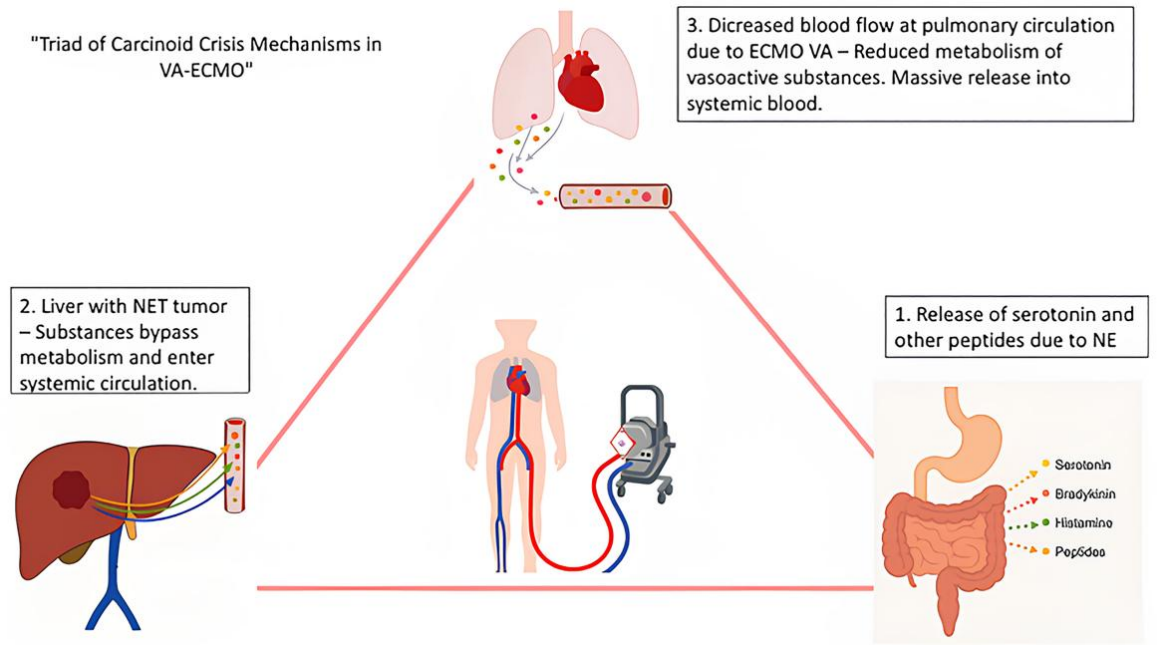
Mechanisms underlying carcinoid crisis in patients with CHD supported with VA-ECMO. Vasoactive substances released from neuroendocrine tumours bypass hepatic and pulmonary metabolism, leading to increased systemic exposure and severe haemodynamic compromise.

## Case presentation

A 52-year-old male with hypertension and dyslipidaemia was admitted due to constitutional symptoms and acute dyspnoea in a tertiary hospital. He reported a 6 kg weight loss over the last week, chronic diarrhoea and recurrent episodes of intense sweating, flushing, and hypertensive crises.

**Figure 1:**
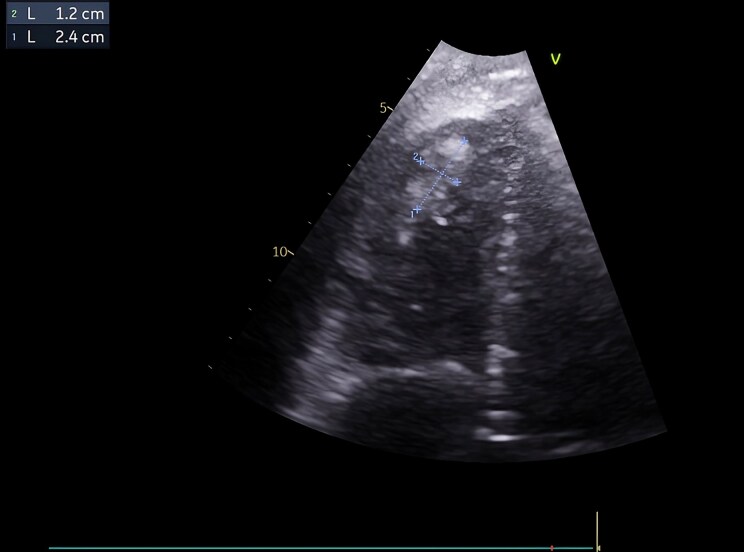
Transthoracic echocardiography, four-chamber view focused on the right ventricle, showing a mass consistent with a thrombus at the right ventricular apex.

On physical examination, he presented with mild hypotension (TAS 95/60 mmHg), tachycardia (101 bpm) and ascites. Laboratory findings revealed multiorgan failure including acute liver injury with coagulopathy and acute kidney injury. Bedside transthoracic echocardiography (TTE) showed preserved left ventricular ejection fraction, right ventricular dilation with mild dysfunction, severe tricuspid regurgitation due to restricted leaflet motion and a mobile mass in right ventricular apex suggestive of thrombus of 24 × 12 mm (*Figure 1* and Supplementary material online, *Videos S1*  to  *S4*). A CT pulmonary angiogram was performed, revealing extensive bilateral pulmonary embolism (see Supplementary material online, *Video S5*).

Given TTE findings CHD was suspected. The patient was transferred to our facility and he rapidly developed haemodynamic deterioration requiring vasopressors, non-invasive mechanical ventilation and continuous renal replacement therapy. Despite optimization of preload, afterload, and inotropic support, the patient progressed to refractory cardiogenic shock (SCAI D) with rising lactate levels and persistent hypotension. At this point, awake VA-ECMO was implanted followed by invasive mechanical ventilation while on support. Patient’s status did not improve in spite of high ECMO flows (4 L/min) and multiple vasopressors (norepinephrine, argipressin, and dobutamine). He also exhibited severe vasoplegia and hypovolemia, requiring aggressive fluid resuscitation. Despite all these therapies lactate levels continued to rise and central venous oxygen saturation (SvO_2_) progressively declined. An abdominal CT scan was performed to rule out other causes of hyperlacticaemia (see Supplementary material online, *Video S6*).

As VA-ECMO support bypasses pulmonary circulation, carcinoid mediators are predominantly released to systemic circulation resulting in severe vasoplegia due to its vasodilatory effects. This situation is known as carcinoid storm. Octreotide and dexchlorpheniramine were started in order to treat carcinoid crisis. Right ventricular thrombus precluded percutaneous right ventricular assist device (RVAD) implantation and the patient finally died 2 days after admission.

His family declined a clinical autopsy but post-mortem laboratory results while on ECMO showed highly elevated chromogranin levels, supporting the diagnosis of CHD.

## Discussion

**Figure 2:**
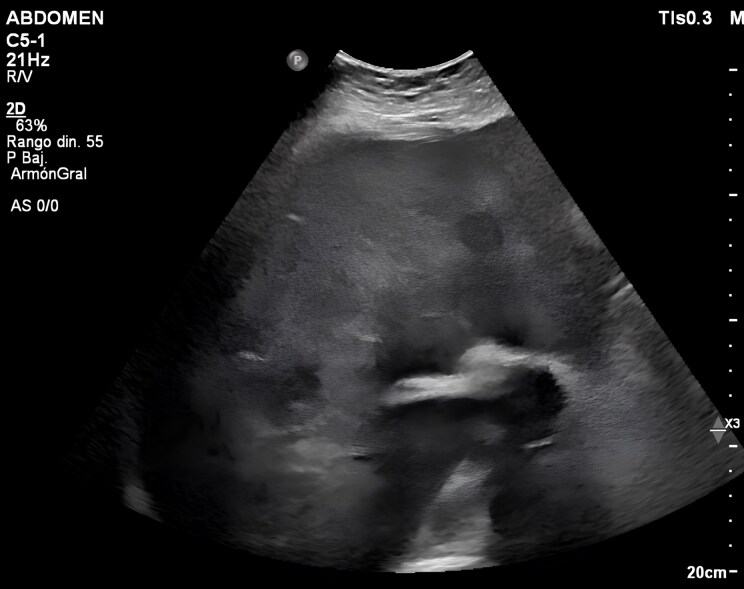
Abdominal ultrasound showing signs of chronic liver disease with multiple hypoechoic hepatic lesions.

CHD (also known as Hedinger syndrome) is an infrequent but serious manifestation of neuroendocrine tumours (NETs). It is characterized by progressive right-sided cardiac involvement due to chronic exposure to vasoactive substances, primarily serotonin, which exert profibrotic effects on the endocardium. These substances are normally metabolized by the liver and lungs. In presence of hepatic metastases hepatic (*Figure 2*) clearance is bypassed, allowing vasoactive agents to reach systemic circulation. This can lead to symptoms such as flushing, diarrhoea, hypotension, and ultimately cardiac involvement.^1^

Cardiac lesions primarily affect tricuspid and pulmonary valves, resulting in leaflet thickening, retraction, and severe regurgitation, which cause right ventricular dilation and eventually right heart failure. In advanced stages, patients may present with low-output syndrome and refractory right-sided heart failure. Surgical valve replacement remains the only definitive therapy for selected patients, though it carries significant operative risk.^2,3^

In critical care scenarios such as acute right ventricular failure, mechanical circulatory support (MCS) may be considered. However, use of VA-ECMO should be avoided in this context. VA-ECMO bypasses pulmonary circulation, the primary site of serotonin metabolism. In patients with hepatic metastases and already compromised liver function, bypassing the lungs further exacerbates systemic exposure to vasoactive substances. As a result, these mediators enter systemic circulation leading to dramatically elevated plasma levels and potentially triggering a fulminant carcinoid crisis or carcinoid storm.

Extracorporeal hemoadsorption techniques are a potential strategy to reduce the systemic effect of vasoactive mediators by removing of circulating cytokines, endotoxins and other mediators.^4^ In our case, continuous veno-venous hemodiafiltration using an oXiris® filter was used for this purpose. Potential impact of hemoadsorption techniques in this context remains unclear and required further investigation.

This storm is characterized by profound haemodynamic instability, refractory vasoplegia, catecholamine-resistant hypotension, and rapid progression to multiorgan failure. This case illustrates catastrophic outcomes after VA-ECMO initiation in patients with carcinoid syndrome and cardiogenic shock. This situation has also been described after cardiac surgery.^2,5^

Carcinoid patients with refractory cardiogenic shock could benefit from MCS devices that preserve pulmonary metabolism, such as RVAD (Protek-Duo or Centrimag or V-PA ECMO) to avoid carcinoid storm.^2,5^ In our case, the presence of a mobile apical thrombus constitutes a major limitation for the use of percutaneous isolated right ventricular mechanical support, further complicating therapeutic decision-making.

Although this entity is well recognized in Cardio-Oncology ESC Guidelines this document is mainly focused on early detection, multidisciplinary evaluation, and timely referral for valve intervention.^6^ However, no specific recommendations are provided regarding use of MCS in the setting of right ventricular failure.

This case highlights the importance of a physiology-based management strategy in CHD with cardiogenic shock. Therapeutic decisions must be guided not only by haemodynamic parameters but also by a thorough understanding of neuroendocrine pathophysiology.

## Supplementary Material

ytag272_Supplementary_Data

## Data Availability

The data underlying this article are not publicly available due to patient privacy, but are available from the corresponding author upon reasonable request.

## Lead author biography

Ana Alicia Alañón Hernández is a cardiology resident at the Department of Cardiology, Hospital Clínico Universitario de Valladolid, Spain. She is currently completing her specialist training in cardiology, with a particular interest in acute cardiac care, mechanical circulatory support, and heart failure. She actively participates in research projects and educational activities within her department and has presented cases and communications at national cardiology congresses.
